# Sufentanil attenuates LPS-induced acute lung injury by suppressing ferroptosis and maintaining GPX4 protein abundance

**DOI:** 10.3389/fcell.2026.1858772

**Published:** 2026-08-21

**Authors:** Xiaoyun Guo, Weifeng Yan, Wenyong Peng, Runbin Yan

**Affiliations:** 1 Department of Anesthesiology, Jinhua Maternal and Child Health Hospital, Jinhua, China; 2 Department of Anestheiology, Jinhua Municipal Central Hospital, Jinhua, China

**Keywords:** acute lung injury, chaperone-mediated autophagy, ferroptosis, GPx4, lipopolysaccharide, sufentanil

## Abstract

**Introduction:**

Acute lung injury (ALI) is a severe inflammatory syndrome characterized by disruption of the alveolar-capillary barrier, pulmonary edema, and impaired gas exchange. Ferroptosis has emerged as an important contributor to ALI pathogenesis, but the mechanisms associated with reduced glutathione peroxidase 4 (GPX4) protein abundance during lung injury remain incompletely understood.

**Methods:**

This study investigated the protective effects of sufentanil pretreatment against lipopolysaccharide (LPS)-induced ALI and examined whether these effects were associated with ferroptosis suppression and maintenance of GPX4 protein abundance. A mouse model of LPS-induced ALI and LPS-stimulated A549 cells were used to evaluate the effects of sufentanil *in vivo* and *in vitro*. The contribution of GPX4 was assessed using siRNA-mediated knockdown and pharmacological modulation of ferroptosis. Potential CMArelated lysosomal involvement was explored using pathway inhibition, coimmunoprecipitation, colocalization analysis, and LAMP2A knockdown.

**Results:**

Sufentanil pretreatment alleviated LPS-induced lung injury, reduced inflammatory responses, and attenuated ferroptosis-associated alterations *in vivo*. In A549 cells, sufentanil reduced mitochondrial and lipid reactive oxygen species, intracellular Fe2+ accumulation, mitochondrial damage, and ACSL4 upregulation, while restoring GPX4 and SLC7A11 protein abundance. GPX4 knockdown markedly attenuated the protective effects of sufentanil. The maintenance of GPX4 protein abundance observed after sufentanil pretreatment was accompanied by reduced GPX4-LAMP2A colocalization and reduced association between GPX4 and HSC70. Furthermore, erastin partially reversed the protective effects of sufentanil *in vivo*.

**Discussions:**

These findings support a preconditioning effect of sufentanil against LPS-induced ALI, associated with ferroptosis suppression, maintenance of GPX4 protein abundance, and possible CMA-related lysosomal involvement.

## 1 Introduction

Acute lung injury (ALI) and its more severe form, acute respiratory distress syndrome (ARDS), remain major challenges in critical care medicine (Matthay et al., 2024). They are characterized by disruption of the alveolar-capillary barrier, diffuse alveolar damage, pulmonary edema, and severe hypoxemia (Ma et al., 2025). Despite advances in supportive care, including lung-protective ventilation and extracorporeal membrane oxygenation, the management of ARDS remains largely supportive, and mortality is still substantial (Qadir et al., 2024; Gorman et al., 2022). Therefore, identifying actionable molecular mechanisms that drive lung injury is essential for the development of more effective therapies.

Among the mechanisms implicated in ALI, ferroptosis has emerged as a relevant form of regulated cell death (Wang X. et al., 2022). Ferroptosis is characterized by iron-dependent lipid peroxidation, depletion of antioxidant defenses, and distinctive mitochondrial abnormalities (Dixon and Olzmann, 2024). Increasing evidence indicates that ferroptosis contributes to pulmonary epithelial and endothelial injury across multiple forms of ALI, including LPS-induced lung injury, sepsis-related ALI, and ischemia-reperfusion-associated injury (Liu et al., 2020; Fan et al., 2025; Wu et al., 2024; Zhang et al., 2023). Experimental studies further show that pharmacological inhibition of ferroptosis alleviates lung inflammation and tissue damage (Lai et al., 2023), supporting the view that ferroptosis is not merely an epiphenomenon but an important pathogenic component of ALI.

Glutathione peroxidase 4 (GPX4) is a central suppressor of ferroptosis. By reducing phospholipid hydroperoxides to less reactive lipid alcohols, GPX4 preserves membrane redox homeostasis and protects cells from lethal lipid peroxidation (Xie et al., 2023; Liu et al., 2023). In the context of ALI, previous studies have mainly focused on transcriptional regulatory pathways upstream of GPX4, such as the Nrf2-centered antioxidant network (Li et al., 2022; Shen et al., 2023). However, transcriptional regulation alone may not fully explain the rapid loss of GPX4 protein under pathological stress. Recent work in other disease settings suggests that GPX4 abundance can also be controlled at the post-translational level (Huang et al., 2025; Wang et al., 2025; Zhu et al., 2017), raising the possibility that disturbed protein homeostasis may critically shape ferroptosis sensitivity in injured lung tissue.

Chaperone-mediated autophagy (CMA) is a selective lysosomal degradation pathway that depends on HSC70-mediated substrate recognition and LAMP2A-dependent lysosomal translocation (Kaushik and Cuervo, 2018; Yao and Shen, 2020). Previous studies in other disease contexts have reported that CMA activation can promote GPX4 degradation and thereby facilitate ferroptotic cell death (Chen et al., 2021; Wu et al., 2019). Subsequent studies in tumor models further suggested that interference with CMA-related GPX4 turnover may help maintain GPX4 protein abundance and attenuate ferroptosis (Chen et al., 2025; Hirata et al., 2024). However, whether CMA or related lysosomal pathways are associated with GPX4 protein loss during ALI, particularly in alveolar epithelial cells, remains to be fully elucidated.

Sufentanil is a widely used opioid analgesic in perioperative and critical care settings (Monk et al., 1988). Beyond its analgesic effect, emerging studies suggest that sufentanil may exert anti-inflammatory and cytoprotective actions in several injury models (Lian et al., 2019; Wang Z. et al., 2022). Recent evidence indicates that sufentanil attenuates septic acute lung injury, whereas studies in cerebral and hepatic ischemia-reperfusion models suggest that its protective effects may be associated with suppression of ferroptosis (Hou et al., 2024; Zhu et al., 2024; Feng et al., 2025). However, whether sufentanil modulates ferroptosis in ALI, and more specifically whether it maintains GPX4 protein abundance in association with CMA-related lysosomal regulation, has not been directly established.

In the present study, we investigated whether sufentanil pretreatment protects against LPS-induced ALI and whether this effect is associated with ferroptosis suppression and maintenance of GPX4 protein abundance. Using *in vivo* and *in vitro* models, we further assessed the contribution of GPX4 to the protective effect of sufentanil and explored whether CMA-related lysosomal processes are associated with reduced GPX4 protein abundance, a relationship that remains largely unexplored in ALI.

## 2 Materials and methods

### 2.1 Reagents and antibodies

Lipopolysaccharide (LPS) from *Escherichia coli* O111:B4 was purchased from Sigma-Aldrich (L2630). Erastin (HY-15763), MG-132 (HY-13259) and Naloxone (HY-17417A) were purchased from MedChemExpress. Chloroquine diphosphate salt was purchased from Sigma-Aldrich (C6628). TRIzol Reagent and Lipofectamine RNAiMAX Transfection Reagent were purchased from Invitrogen (15596026 and 13778075, respectively). BODIPY 581/591 C11, MitoSOX Red, and DAPI were purchased from Invitrogen (D3861, M36008, and D1306, respectively). FerroOrange, Cell Counting Kit-8, GSSG/GSH Quantification Kit, and MDA Assay Kit were purchased from Dojindo (F374, CK04, G257, and M496, respectively). A549 cells were obtained from ATCC (CCL-185). BALF total protein was measured using Protein Assay Dye Reagent Concentrate (Bio-Rad, 5000006). Fe^2+^ content in tissue homogenates was measured using Iron Assay Kit (Sigma-Aldrich, MAK025). BALF concentrations of TNF-α, IL-6, and IL-1β were determined using ELISA kits (Abcam, ab208348, ab222503, and ab197742, respectively). Reverse transcription was performed using High-Capacity cDNA Reverse Transcription Kit (Applied Biosystems, 4368814), and quantitative PCR was performed using PowerUp SYBR Green Master Mix (Applied Biosystems, A25742). MPO activity was measured using a commercial assay kit from Nanjing Jiancheng Bioengineering Institute (A044-1-1). Sufentanil citrate injection was diluted in sterile saline before use.

Primary antibodies used in this study were as follows: anti-GPX4 (Abcam, ab125066), anti-ACSL4 (Cell Signaling Technology, #38493), anti-xCT/SLC7A11 (Cell Signaling Technology, #12691), anti-LAMP2A (Abcam, ab125068), anti-HSC70/HSPA8 (Cell Signaling Technology, #8444), anti-4-hydroxynonenal (4-HNE) (Abcam, ab48506), and anti-GAPDH (Cell Signaling Technology, #5174). HRP-linked anti-rabbit IgG and anti-mouse IgG secondary antibodies for Western blotting were from Cell Signaling Technology (#7074 and #7076, respectively). Alexa Fluor 488 goat anti-rabbit IgG and Alexa Fluor 594 goat anti-mouse IgG secondary antibodies for immunofluorescence were from Invitrogen (A-11008 and A-11032, respectively).

### 2.2 Animals

Male C57BL/6 mice aged 8–10 weeks and weighing 20–24 g were obtained from Cyagen Biosciences Inc. (Suzhou, China). Mice were housed under specific pathogen-free conditions at 22 °C ± 2 °C, with a 12-h light/dark cycle, 50%–60% relative humidity, and free access to food and water. All mice were acclimatized for at least 1 week before the experiments. The study protocol was approved by the Laboratory Animal Welfare and Ethics Committee of Jinhua Municipal Central Hospital(NO.AL-JHYY202633). All animal experiments were performed in accordance with institutional guidelines and applicable national regulations governing the care and use of laboratory animals.

### 2.3 LPS-induced ALI mouse model

Male C57BL/6 mice were randomly assigned to the experimental groups. For the primary ALI experiment, mice were assigned to the Sham, LPS, and LPS + Suf groups, with five mice per group. For the *in vivo* pharmacological reversal experiment, mice were assigned to the Sham, LPS, LPS + Suf, and LPS + Suf + erastin groups, with five mice per group. Mice were anesthetized with pentobarbital sodium, and ALI was induced by intratracheal instillation of LPS at 10 mg/kg in a total volume of 50 μL sterile saline. Animals in the Sham group received an equivalent volume of sterile saline. Sufentanil was administered through the tail vein at 3 μg/kg 30 min before LPS challenge. To determine whether ferroptosis contributed to the protective effects of sufentanil, erastin was used as a pharmacological inducer of ferroptosis. Erastin was administered intraperitoneally at 15 mg/kg 30 min before sufentanil administration. Erastin was used to enhance ferroptosis and functionally challenge the anti-ferroptotic effect of sufentanil, rather than as a direct or selective inhibitor of GPX4. At 24 h after LPS challenge, mice were anesthetized using 0.3% pentobarbital sodium at 200 μL per 10 g body weight and euthanized by cervical dislocation. BALF and lung tissues were subsequently collected for analysis.

### 2.4 Histopathological examination

**FIGURE 1:**
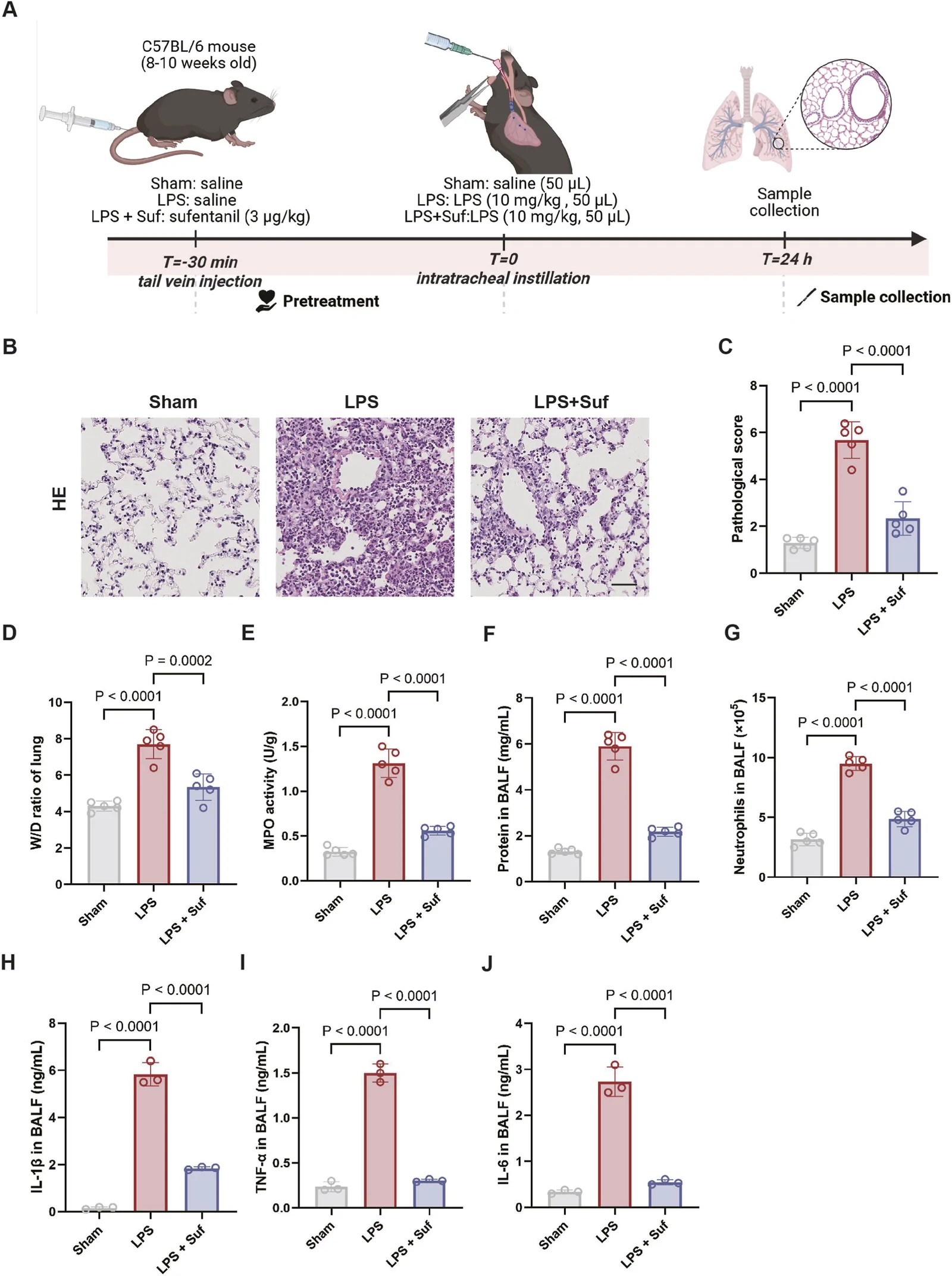
Sufentanil pretreatment attenuates LPS-induced acute lung injury in mice **(A)** Schematic illustration of the experimental design. **(B)** Representative H&E-stained lung sections from the Sham, LPS and LPS + Suf groups. Scale bar, 50 µm. **(C)** Semiquantitative pathological score of lung injury. **(D)** Lung wet-to-dry (W/D) weight ratio. **(E)** Myeloperoxidase (MPO) activity in lung tissue. **(F)** Total protein concentration in bronchoalveolar lavage fluid (BALF). **(G)** Neutrophil counts in BALF. **(H–J)** BALF concentrations of IL-1β **(H)**, TNF-α **(I)** and IL-6 **(J)**. Data are presented as mean ± SD. Each dot represents one biologically independent mouse (n = 5 per group). Statistical significance was determined by one-way ANOVA followed by Tukey’s multiple-comparisons test. Exact P values are shown in the figure.

Lung tissues were fixed in 4% paraformaldehyde, embedded in paraffin, and sectioned at 3–5 μm thickness. Sections were stained with hematoxylin and eosin (H&E) to assess histopathological changes. Lung injury was evaluated using a modified semiquantitative scoring system by two investigators blinded to group allocation. Four pathological parameters were assessed: alveolar congestion, hemorrhage, neutrophil infiltration or aggregation in the airspace or vessel wall, and alveolar wall thickening/hyaline membrane formation. Each parameter was scored on a scale of 0–4, and the total score was used to evaluate the severity of lung injury. H&E staining was used in Figure 1 for baseline injury evaluation and in Figure 6 for rescue validation.

### 2.5 Immunohistochemistry

Immunohistochemistry was performed on paraffin-embedded lung sections following deparaffinization, rehydration, antigen retrieval, endogenous peroxidase blocking, and serum blocking. Sections were incubated overnight at 4 °C with primary antibodies against GPX4 or 4-HNE, followed by incubation with the appropriate HRP-conjugated secondary antibodies and visualization using 3,3′-diaminobenzidine. Nuclei were counterstained with hematoxylin. Images were acquired using identical magnification, illumination, and exposure settings for all groups within the same experiment. For each animal, five randomly selected nonoverlapping fields were analyzed. Positive staining was quantified using ImageJ software with identical thresholding and analysis settings across all groups. Quantification was performed by an investigator blinded to group allocation. Values obtained from multiple fields of the same animal were averaged, and each animal was considered one biological replicate.

### 2.6 BALF collection and inflammatory cytokine measurement

BALF was collected through tracheal cannulation by flushing the lungs three times with 0.5 mL ice-cold PBS. The recovered volume was recorded, and samples with a recovery rate below 80% were excluded according to a prespecified criterion. BALF was centrifuged at 500 *g* for 10 min at 4 °C. The supernatant was collected for total protein and cytokine measurements, and the cell pellet was resuspended in PBS for total and differential cell counting. Neutrophil counts were determined using Wright–Giemsa-stained smears by counting at least 200 cells per sample. BALF total protein was measured using Protein Assay Dye Reagent Concentrate (Bio-Rad, 5000006). BALF concentrations of TNF-α, IL-6, and IL-1β were measured using ELISA kits from Abcam (ab208348, ab222503, and ab197742, respectively), according to the manufacturer’s instructions. Each sample was measured in triplicate, and technical replicate values were averaged before statistical analysis.

### 2.7 Lung wet-to-dry weight ratio and biochemical assays

Fresh lung tissue was weighed immediately after collection to obtain the wet weight and then dried in an oven at 60 °C until a constant weight was reached. The wet-to-dry weight ratio was calculated by dividing the wet weight by the dry weight. Lung tissues were homogenized in ice-cold PBS or the assay buffer provided with each kit and centrifuged at 12,000 × g for 10 min at 4 °C. The resulting supernatants were used for biochemical assays. MDA and GSH/GSSG levels were measured using the MDA Assay Kit and GSSG/GSH Quantification Kit from Dojindo (M496 and G257, respectively). Fe^2+^ levels were measured using the Iron Assay Kit from Sigma-Aldrich (MAK025). Assays were performed according to the manufacturers’ instructions. Fe^2+^ and MDA levels were normalized to total protein concentration, and GSH/GSSG was calculated as a ratio.

### 2.8 MPO activity assay

Myeloperoxidase (MPO) activity in lung tissues was measured using a commercial MPO assay kit (Nanjing Jiancheng Bioengineering Institute, A044-1-1) according to the manufacturer’s instructions. Briefly, lung tissues were homogenized in the assay buffer provided with the kit and centrifuged to remove insoluble material. The supernatant was collected for colorimetric detection. Absorbance was measured at 460 nm using a microplate reader. MPO activity was normalized to lung tissue wet weight and expressed as U/g tissue.

### 2.9 Cell lines and culture

A549 cells were obtained from ATCC (CCL-185) and cultured in F-12K medium supplemented with 10% fetal bovine serum and 1% penicillin/streptomycin at 37 °C in a humidified atmosphere containing 5% CO_2_.

### 2.10 Cell treatment

To establish the *in vitro* inflammatory injury model, A549 cells were exposed to LPS at 10 μg/mL for 24 h. For dose-screening experiments, cells were pretreated with sufentanil at 5, 10, 20, 40, or 60 μM for 1 h before LPS exposure. Based on the dose-screening experiment, 40 μM sufentanil was used for subsequent *in vitro* experiments. To evaluate opioid receptor involvement, cells were pre-incubated with the non-selective opioid receptor antagonist naloxone at 10 μM for 30 min before sufentanil treatment. To examine the possible involvement of lysosomal and proteasomal pathways in the regulation of GPX4 protein abundance, cells were treated with chloroquine at 20 μM or MG-132 at 10 μM during the final 6 h before cell collection. For the 24-h LPS experiments, chloroquine or MG-132 was therefore added during the final 6 h of the treatment period. Corresponding control groups received an equivalent volume of solvent.

### 2.11 Cell viability assay

Cells were seeded into 96-well plates at 5 × 10^3^ to 6 × 10^3^ cells per well and treated as indicated. Cell viability was measured using the Cell Counting Kit-8, with absorbance read at 450 nm using a microplate reader. Each condition contained three technical replicate wells in each independent experiment. Technical replicate values were averaged to generate one value for each biological replicate.

### 2.12 Lipid ROS, mitochondrial ROS, and intracellular Fe^2+^ assays

For lipid ROS measurement, cells were incubated with BODIPY 581/591 C11 at 5 μM for 30 min. For Fe^2+^ detection, cells were incubated with FerroOrange according to the manufacturer’s protocol. For mitochondrial oxidative stress analyses, MitoSOX Red was used according to the manufacturers’ instructions. For flow-cytometric analyses, at least 10,000 events per sample were acquired after exclusion of debris and doublets. Identical gating strategies were applied to all groups within the same experiment. Flow-cytometric data were analyzed by an investigator blinded to treatment allocation. One independently cultured and treated cell preparation was considered one biological replicate. Multiple flow-cytometric measurements from the same preparation were considered technical replicates and were averaged before statistical analysis. Experiments were repeated independently three times.

### 2.13 RNA extraction and quantitative real-time PCR

Total RNA was extracted using TRIzol Reagent and reverse-transcribed into cDNA using the High-Capacity cDNA Reverse Transcription Kit. Quantitative PCR was performed using PowerUp SYBR Green Master Mix, and relative mRNA expression was calculated using the 2^−ΔΔCT^ method with GAPDH as the internal control. The primer sequences used for A549 cells were as follows: GPX4 forward, 5′-TCA​GCA​AGA​TCT​GCG​TGA​AC-3′ and reverse, 5′-ATA​GTG​GGG​CAG​GTC​CTT​CT-3′; GAPDH forward, 5′-GTC​AGT​GGT​GGA​CCT​GAC​CT-3′ and reverse, 5′-TGC​TGT​AGC​CAA​ATT​CGT​TG-3′.

### 2.14 siRNA transfection and rescue assays

Validated siRNAs targeting human GPX4 and LAMP2A together with a non-targeting scrambled negative control siRNA, were synthesized by GenePharma (Shanghai, China). The siRNA sequences are listed in Supplementary Table S1. Cells were transfected using Lipofectamine RNAiMAX according to the manufacturer’s instructions. The siRNA sequences are listed in Supplementary Table S1. Each experiment was repeated independently three times. After 36–48 h, cells were subjected to LPS with or without sufentanil, and rescue effects were evaluated by cell viability, lipid ROS, Fe^2+^, Western blotting, and colocalization assays.

### 2.15 Western blotting and densitometric analysis

Cells or lung tissues were lysed in RIPA lysis buffer supplemented with protease inhibitors. Equal amounts of protein were separated by SDS-PAGE and transferred onto PVDF membranes. Membranes were blocked with 5% non-fat milk and incubated overnight at 4 °C with the indicated primary antibodies, followed by incubation with HRP-linked secondary antibodies from Cell Signaling Technology (#7074 or #7076). Protein bands were visualized using enhanced chemiluminescence and captured using a chemiluminescence imaging system. Densitometric analysis was performed using ImageJ software on uncropped images acquired within the linear exposure range. Target protein signals were normalized to GAPDH, and the normalized value of the corresponding control group was set to 1. Data are expressed as relative protein abundance. Western blot densitometry was performed by an investigator blinded to group allocation. All western blots used to support quantitative conclusions were quantified from independent biological experiments, as indicated in the corresponding figure legends.

### 2.16 Co-immunoprecipitation and colocalization analysis

For co-immunoprecipitation, cell lysates were incubated with anti-GPX4 antibody (Abcam, ab125066), and the immunoprecipitated proteins were analyzed by Western blotting using anti-HSC70/HSPA8 antibody (Cell Signaling Technology, #8444). Species-matched IgG and input lysates were included as controls. Where quantified, the HSC70 signal detected in the GPX4 immunoprecipitate was normalized to the amount of immunoprecipitated GPX4. For colocalization analysis, cells were stained with antibodies against GPX4 and LAMP2A, followed by Alexa Fluor 488 goat anti-rabbit IgG and Alexa Fluor 594 goat anti-mouse IgG secondary antibodies. Nuclei were counterstained with DAPI. Images were acquired using confocal microscopy under identical acquisition settings for all groups. For each biological replicate, at least 30 cells from randomly selected fields were analyzed. GPX4–LAMP2A colocalization was quantified using Pearson’s correlation coefficient. Values from multiple cells in the same independently treated cell preparation were averaged to obtain one biological replicate. Image acquisition and colocalization analysis were performed by investigators blinded to treatment allocation. Co-immunoprecipitation and colocalization experiments were repeated independently three times.

### 2.17 Statistical analysis

Data are presented as mean ± SD unless otherwise indicated. Biological replicates were defined as individual mice for *in vivo* experiments and as independent cell-culture experiments performed on different days for *in vitro* experiments. Technical replicates were averaged before statistical analysis and were not treated as independent biological replicates. The number of biological replicates or independent experiments is indicated in each figure legend. Normality was assessed using the Shapiro-Wilk test, and homogeneity of variance was assessed using Levene’s test or Brown-Forsythe test. For comparisons among more than two groups, one-way ANOVA followed by Tukey’s multiple-comparisons test was used when assumptions were met. For experiments involving two independent factors, such as siRNA treatment and sufentanil exposure, two-way ANOVA followed by appropriate multiple-comparisons testing was used to evaluate main effects and interactions. If data did not meet parametric assumptions, the Kruskal–Wallis test followed by Dunn’s multiple-comparisons test was used. Statistical analyses were performed using GraphPad Prism 9.5. A value of P < 0.05 was considered statistically significant.

## 3 Results

### 3.1 Sufentanil pretreatment attenuates LPS-induced acute lung injury in mice

To evaluate the protective effect of sufentanil pretreatment against LPS-induced ALI, we established an LPS-induced murine ALI model and assessed histological and inflammatory changes 24 h after LPS challenge (Figure 1A). H&E staining showed that LPS administration caused disruption of the alveolar architecture, inflammatory cell infiltration, alveolar wall thickening, and narrowing of the alveolar spaces compared with the Sham group (Figure 1B). These histopathological changes were attenuated by sufentanil pretreatment, as further demonstrated by the reduced lung injury score (Figure 1C). Consistent with the histological findings, LPS challenge significantly increased the lung wet-to-dry weight ratio, indicating pulmonary edema, whereas sufentanil pretreatment reduced this increase (Figure 1D). Lung myeloperoxidase activity, an indicator of neutrophil accumulation, was also elevated after LPS exposure and reduced by sufentanil pretreatment (Figure 1E). In parallel, LPS increased the total protein concentration and neutrophil count in bronchoalveolar lavage fluid (BALF), indicating alveolar-capillary barrier disruption and inflammatory cell recruitment. Both changes were attenuated by sufentanil pretreatment (Figures 1F,G).

LPS challenge also increased the BALF concentrations of IL-1β, TNF-α, and IL-6, whereas sufentanil pretreatment significantly reduced all three cytokines (Figures 1H–J). Together, these findings indicate that sufentanil pretreatment attenuates LPS-induced ALI, as evidenced by reduced histopathological injury, pulmonary edema, alveolar-capillary barrier disruption, neutrophil accumulation, and inflammatory cytokine production.

### 3.2 Sufentanil pretreatment attenuates ferroptosis-associated changes in LPS-induced ALI

**FIGURE 2:**
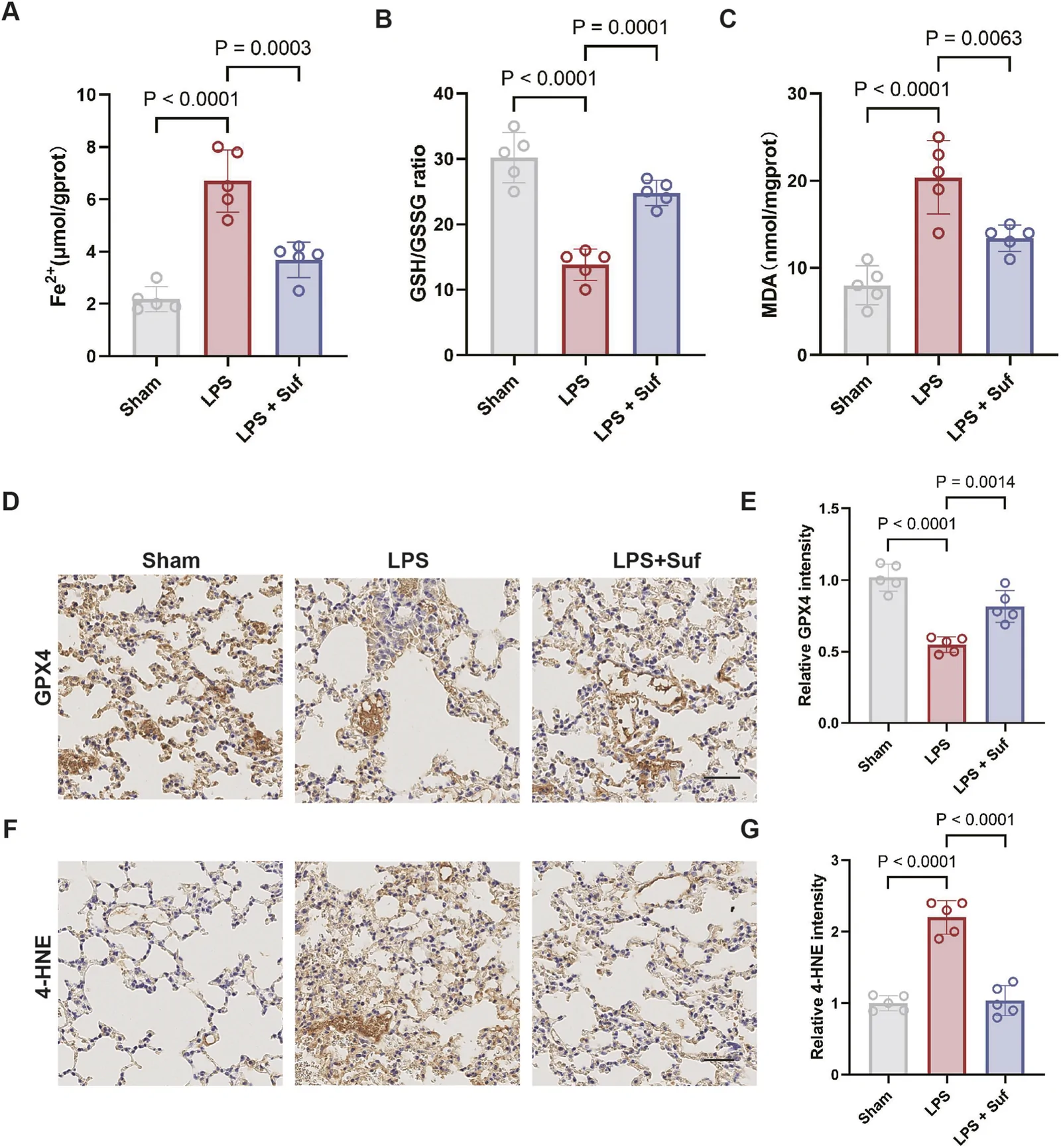
Sufentanil pretreatment attenuates ferroptosis-associated oxidative alterations and restores GPX4 immunoreactivity in LPS-induced acute lung injury **(A)** Fe^2+^ levels in lung tissues from the Sham, LPS, and LPS + Suf groups. **(B)** GSH/GSSG ratio in lung tissues. **(C)** MDA levels in lung tissues. **(D)** Representative immunohistochemical staining of GPX4 in lung sections from the indicated groups. Scale bar, 50 µm. **(E)** Quantification of relative GPX4 staining intensity. **(F)** Representative immunohistochemical staining of 4-HNE in lung sections from the indicated groups. **(G)**, Quantification of relative 4-HNE staining intensity. Data are presented as mean ± SD. Statistical significance was determined by one-way ANOVA followed by Tukey’s multiple-comparisons test. Exact P values are shown in the figure.

Given the protective effects of sufentanil pretreatment against LPS-induced lung injury, we next examined whether these effects were accompanied by changes in ferroptosis-associated indices *in vivo*. LPS administration significantly increased Fe^2+^ levels in lung tissues, whereas sufentanil pretreatment attenuated this increase (Figure 2A). In parallel, the GSH/GSSG ratio was significantly decreased after LPS challenge, indicating impaired redox homeostasis, and was partially restored by sufentanil pretreatment (Figure 2B). LPS exposure also increased pulmonary MDA levels, consistent with enhanced lipid peroxidation, whereas sufentanil pretreatment significantly reduced this increase (Figure 2C). Immunohistochemical analysis further showed that GPX4 immunoreactivity was markedly reduced in lung tissues after LPS exposure, whereas sufentanil pretreatment partially restored GPX4 staining intensity (Figures 2D,E). Conversely, 4-HNE immunoreactivity was increased after LPS challenge and was significantly reduced by sufentanil pretreatment (Figures 2F,G).

These observations indicate that the protective effects of sufentanil pretreatment were accompanied by reduced iron accumulation and lipid peroxidation, improved redox balance, and partial restoration of GPX4 immunoreactivity in LPS-induced ALI.

### 3.3 Sufentanil pretreatment attenuates ferroptosis-associated changes in LPS-stimulated A549 cells

**FIGURE 3:**
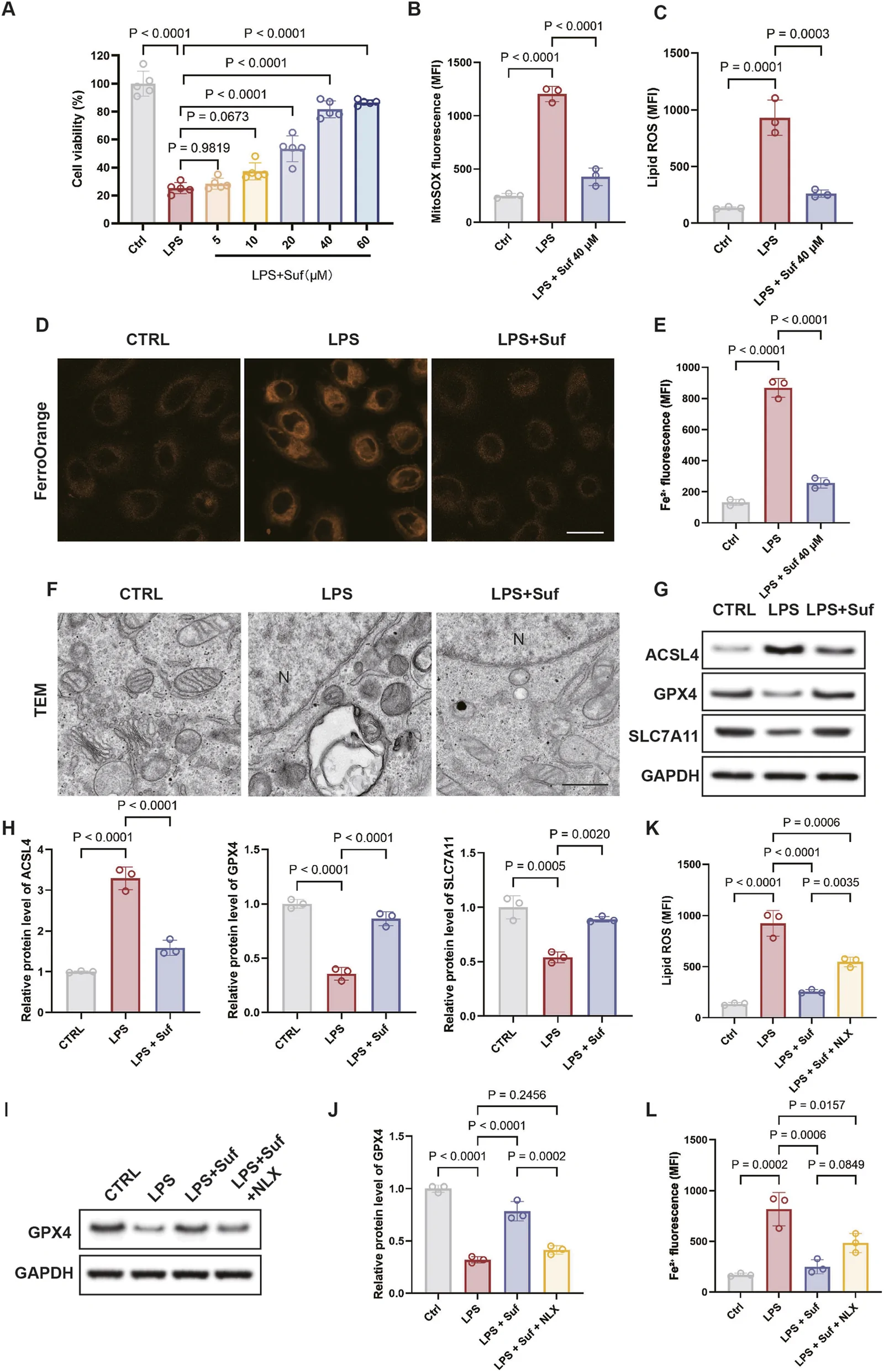
Sufentanil pretreatment attenuates ferroptosis-associated alterations in LPS-stimulated A549 cells **(A)** Cell viability of A549 cells treated with LPS in the absence or presence of increasing concentrations of sufentanil. **(B)** Quantification of MitoSOX fluorescence in the Ctrl, LPS, and LPS + Suf groups by flow cytometry. **(C)** Quantification of lipid ROS levels in the Ctrl, LPS, and LPS + Suf groups. **(D)** Representative FerroOrange fluorescence images showing intracellular Fe^2+^ in the indicated groups. Scale bar, 20 µm. **(E)** Quantification of Fe^2+^ fluorescence intensity. **(F)** Representative transmission electron microscopy images of A549 cells from the indicated groups. Scale bar, 500 nm. **(G)** Representative western blots showing ACSL4, GPX4, and SLC7A11 expression in the indicated groups. **(H)** Densitometric quantification of ACSL4, GPX4, and SLC7A11 protein abundance normalized to GAPDH. **(I)** Representative western blots showing GPX4 and GAPDH expression in cells treated with or without the opioid receptor antagonist naloxone (NLX). **(J)** Densitometric quantification of GPX4 protein abundance normalized to GAPDH. **(K)** Quantification of lipid ROS levels in the presence or absence of NLX. **(L)** Quantification of Fe^2+^ fluorescence intensity in the presence or absence of NLX. Data are presented as mean ± SD. Statistical significance was determined by one-way ANOVA followed by Tukey’s multiple-comparisons test. Exact P values are shown in the figure.

We next examined whether the ferroptosis-associated alterations observed *in vivo* were also present in LPS-stimulated A549 cells and whether they were affected by sufentanil pretreatment. Dose-screening experiments showed that sufentanil increased the viability of LPS-exposed A549 cells in a concentration-dependent manner. A concentration of 40 μM was selected as the experimental proof-of-concept concentration for subsequent analyses because it produced a clear protective effect, whereas increasing the concentration to 60 μM provided no evident additional benefit (Figure 3A).

MitoSOX analysis showed that LPS stimulation significantly increased mitochondrial ROS levels, whereas sufentanil pretreatment attenuated this increase (Figure 3B). Similarly, lipid ROS levels were elevated after LPS exposure and were significantly reduced by sufentanil pretreatment (Figure 3C). FerroOrange staining further demonstrated increased intracellular Fe^2+^ accumulation in LPS-stimulated cells, which was attenuated by sufentanil pretreatment (Figures 3D,E).

Transmission electron microscopy showed that LPS exposure induced mitochondrial shrinkage, increased mitochondrial membrane density, and reduced mitochondrial cristae. These ultrastructural abnormalities were partially alleviated by sufentanil pretreatment (Figure 3F). Representative western blots and corresponding densitometric analyses showed that LPS increased ACSL4 protein abundance while reducing GPX4 and SLC7A11 protein abundance. These changes were partially reversed by sufentanil pretreatment (Figures 3G,H).

To assess the involvement of opioid receptor signaling, cells were pretreated with naloxone, a nonselective opioid receptor antagonist. Western blotting and densitometric analysis showed that naloxone partially attenuated the sufentanil-associated restoration of GPX4 protein abundance (Figures 3I,J). Naloxone also partially reversed the reductions in lipid ROS and intracellular Fe^2+^ observed after sufentanil pretreatment (Figures 3K,L).

Collectively, these findings indicate that sufentanil pretreatment attenuated ferroptosis-associated oxidative, mitochondrial, and molecular alterations in LPS-stimulated A549 cells. The partial reversal observed after naloxone treatment supports the involvement of opioid receptor signaling but does not establish dependence on a specific opioid receptor subtype.

### 3.4 GPX4 knockdown attenuates sufentanil-mediated protection in LPS-stimulated A549 cells

**FIGURE 4:**
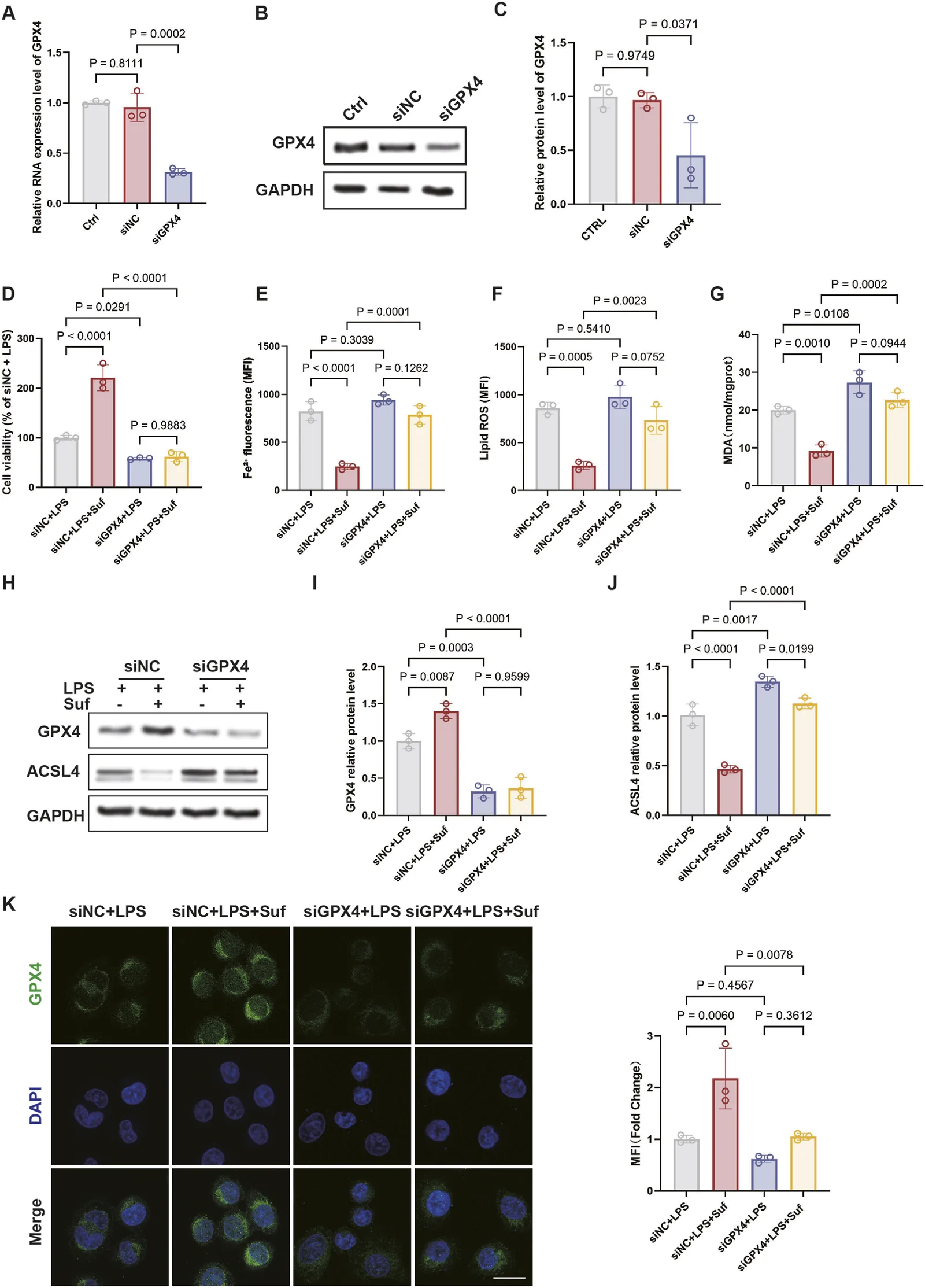
GPX4 knockdown attenuates the protective effect of sufentanil in LPS-treated A549 cells **(A)** Relative GPX4 mRNA expression in Ctrl, siNC, and siGPX4-transfected A549 cells. **(B)** Representative western blots showing GPX4 and GAPDH expression in Ctrl, siNC, and siGPX4-transfected A549 cells. **(C)** Densitometric quantification of GPX4 protein abundance normalized to GAPDH. **(D)** Cell viability in the siNC + LPS, siNC + LPS + Suf, siGPX4 + LPS, and siGPX4 + LPS + Suf groups. **(E)** Quantification of Fe^2+^ fluorescence intensity in the indicated groups. **(F)** Quantification of lipid ROS levels in the indicated groups. **(G)** MDA levels in the indicated groups. **(H)** Representative western blots showing GPX4 and ACSL4 expression in the indicated groups. **(I)** Quantification of relative GPX4 protein levels. **(J)** Quantification of relative ACSL4 protein levels. **(K)** Representative GPX4 immunofluorescence images and quantification of relative GPX4 fluorescence intensity in the indicated groups. Nuclei were counterstained with DAPI. Scale bar, 20 µm. Data are presented as the mean ± SD. Each dot represents one independent biological experiment. Data in **(A–C)** were analyzed using one-way ANOVA followed by Tukey’s multiple-comparisons test. Data in **(D–K)** were analyzed using two-way ANOVA followed by Šídák’s multiple-comparisons test. Exact P values are shown in the figure.

Given that sufentanil pretreatment restored GPX4 protein abundance and attenuated ferroptosis-associated alterations, we next assessed the contribution of GPX4 to its protective effects in LPS-stimulated A549 cells. GPX4 expression was reduced using siRNA, and cell viability, oxidative stress, intracellular iron accumulation, and ferroptosis-related molecular changes were subsequently evaluated. GPX4 knockdown efficiency was first confirmed at both the mRNA and protein levels. GPX4 mRNA expression was significantly reduced in siGPX4-transfected cells, whereas transfection with the negative-control siRNA had no significant effect compared with untreated cells (Figure 4A). Western blotting and densitometric analysis further confirmed reduced GPX4 protein abundance after siGPX4 transfection (Figures 4B,C). In siNC-transfected cells, sufentanil pretreatment significantly increased cell viability under LPS stimulation. This protective effect was markedly attenuated following GPX4 knockdown (Figure 4D). Similarly, sufentanil reduced intracellular Fe^2+^ accumulation, lipid ROS levels, and MDA production in siNC-transfected cells, whereas these effects were substantially weakened in siGPX4-transfected cells (Figures 4E–G). Western blotting further showed that sufentanil increased GPX4 protein abundance and reduced ACSL4 protein abundance in siNC-transfected cells. Following GPX4 knockdown, these effects were markedly attenuated (Figures 4H–J). Consistently, immunofluorescence analysis showed increased GPX4 staining after sufentanil pretreatment in siNC-transfected cells, whereas this increase was diminished in siGPX4-transfected cells (Figure 4K). Collectively, these findings indicate that GPX4 contributes substantially to the protective effects of sufentanil against ferroptosis-associated injury in LPS-stimulated A549 cells.

### 3.5 Sufentanil maintains GPX4 protein abundance in association with CMA-related lysosomal changes

**FIGURE 5:**
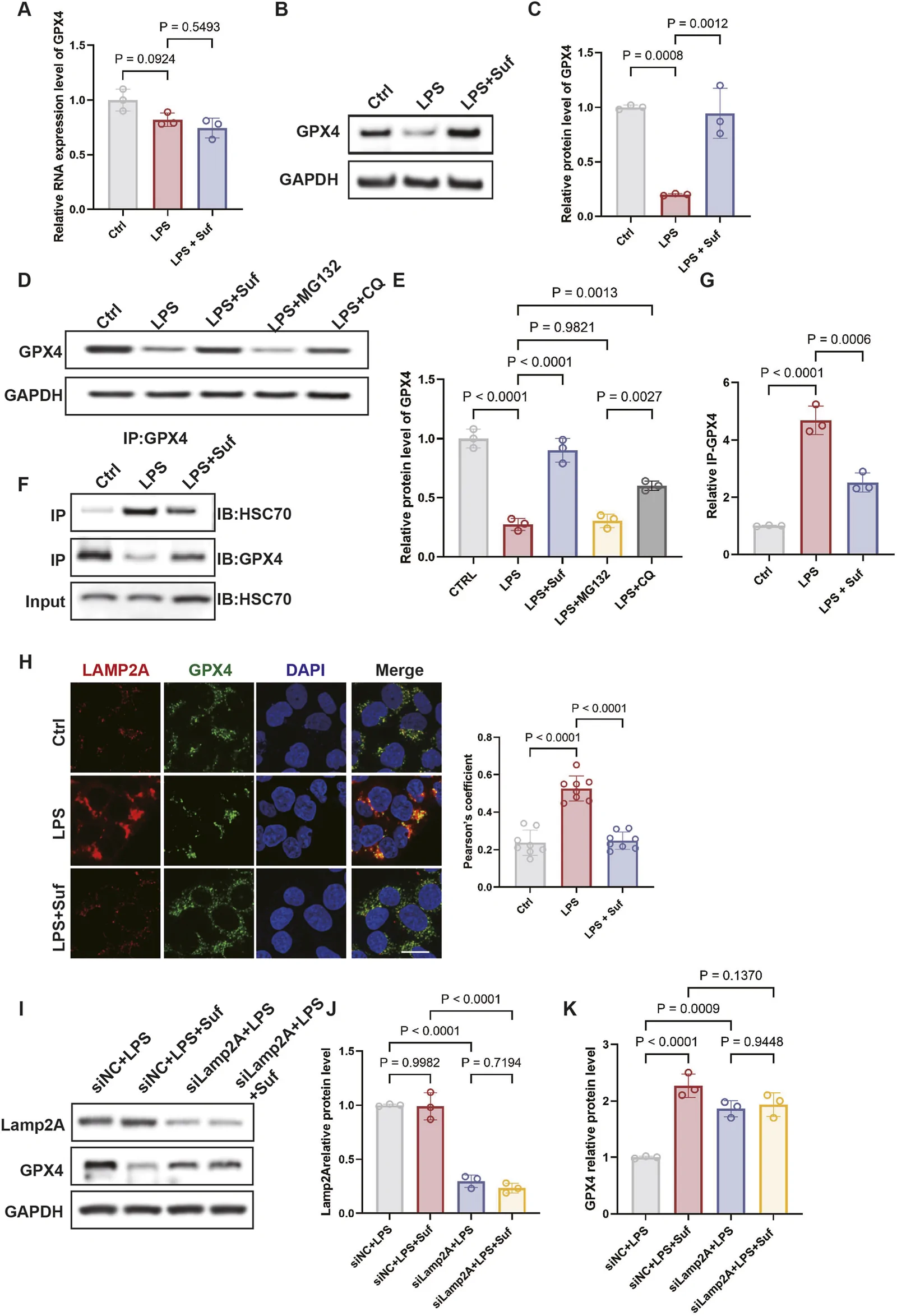
CMA-related lysosomal changes are associated with GPX4 protein abundance in LPS-treated A549 cells **(A)** Relative GPX4 mRNA expression in the Ctrl, LPS, and LPS + Suf groups. **(B)** Representative western blots showing GPX4 and GAPDH expression in the indicated groups. **(C)** Quantification of relative GPX4 protein levels. **(D)** Representative western blots showing GPX4 and GAPDH expression in the Ctrl, LPS, LPS + Suf, LPS + MG132, and LPS + CQ groups. **(E)** Quantification of relative GPX4 protein abundance in the indicated groups. **(F)** Representative co-immunoprecipitation analysis of the interaction between GPX4 and HSC70 in the indicated groups. Cell lysates were immunoprecipitated with anti-GPX4 antibody and immunoblotted for HSC70 or GPX4; input HSC70 is shown below. **(G)** Quantification of relative GPX4–HSC70 interaction. **(H)** Representative immunofluorescence images showing LAMP2A, GPX4, DAPI, and merged signals in the indicated groups, with quantification of GPX4–LAMP2A colocalization using Pearson’s correlation coefficient. Scale bar, 20 μm. **(I)** Representative western blots showing LAMP2A, GPX4, and GAPDH expression in A549 cells transfected with siNC or siLAMP2A in the presence of LPS or LPS + Suf. **(J)** Quantification of relative LAMP2A protein levels. **(K)** Quantification of relative GPX4 protein levels. Data are presented as mean ± SD. Statistical significance was determined by one-way ANOVA followed by Tukey’s multiple-comparisons test. Exact P values are shown in the figure.

We next examined whether the maintenance of GPX4 protein abundance after sufentanil pretreatment was associated with CMA-related lysosomal processes in LPS-stimulated A549 cells. GPX4 mRNA expression did not differ significantly among the Ctrl, LPS, and LPS + Suf groups (Figure 5A). In contrast, Western blotting and densitometric analysis showed that LPS stimulation reduced GPX4 protein abundance, whereas sufentanil pretreatment partially restored it (Figures 5B,C). These findings indicate that the observed changes in GPX4 protein abundance occurred without corresponding detectable changes in GPX4 mRNA expression. To explore the possible involvement of lysosomal and proteasomal pathways, we examined the effects of chloroquine and MG-132 on GPX4 protein abundance. Chloroquine increased GPX4 protein abundance in LPS-stimulated cells, whereas MG-132 produced a less pronounced effect (Figures 5D,E). These results suggest that the reduction in GPX4 protein abundance under LPS stimulation may be more closely associated with lysosomal processes than with proteasomal degradation. However, these inhibitor experiments alone do not establish a specific GPX4 degradation pathway.

We next assessed CMA-related molecular changes. Immunofluorescence analysis showed that GPX4–LAMP2A colocalization was increased after LPS stimulation and reduced following sufentanil pretreatment (Figures 5H,I). Co-immunoprecipitation analysis further showed increased association between GPX4 and HSC70 after LPS stimulation, whereas this association was attenuated by sufentanil pretreatment (Figures 5F,G). To further evaluate the involvement of CMA-related machinery, LAMP2A was silenced using siRNA. LAMP2A knockdown was confirmed by Western blotting and was accompanied by partial restoration of GPX4 protein abundance under LPS stimulation (Figures 5J,K).

Overall, these pharmacological, colocalization, co-immunoprecipitation, and knockdown findings support an association between CMA-related lysosomal processes and reduced GPX4 protein abundance during LPS-induced injury. Sufentanil pretreatment maintained GPX4 protein abundance in association with reduced GPX4–LAMP2A colocalization and GPX4–HSC70 association. However, these findings do not establish GPX4 as a direct CMA substrate or demonstrate definitive CMA-mediated GPX4 degradation.

### 3.6 Erastin partially reverses the protective effects of sufentanil in LPS-induced ALI

**FIGURE 6:**
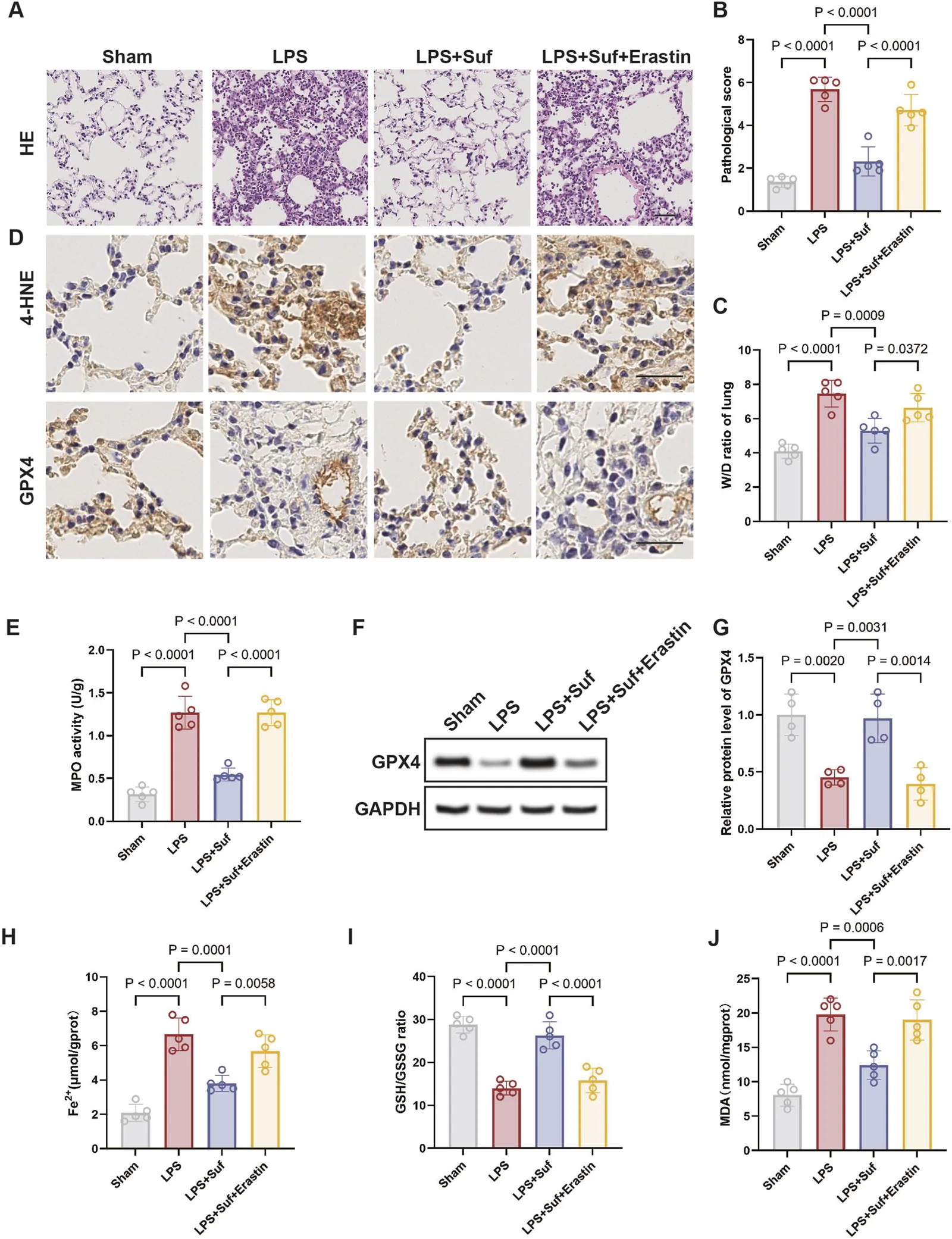
*In vivo* rescue validation with erastin in LPS-induced acute lung injury **(A)** Representative H&E-stained lung sections from the Sham, LPS, LPS + Suf, and LPS + Suf + Erastin groups. Scale bar, 50 µm. **(B)** Semiquantitative pathological score of lung injury in the indicated groups. **(C)** Lung wet-to-dry (W/D) weight ratio in the indicated groups. **(D)** Representative immunohistochemical staining of 4-HNE and GPX4 in lung sections from the indicated groups. Scale bar, 50 µm. **(E)** Myeloperoxidase (MPO) activity in lung tissues from the indicated groups. **(F)** Representative western blots showing GPX4 and GAPDH expression in lung tissues from the indicated groups. **(G)** Quantification of relative GPX4 protein levels. **(H)** Fe^2+^ levels in lung tissues from the indicated groups. **(I)** GSH/GSSG ratio in lung tissues from the indicated groups. **(J)** MDA levels in lung tissues from the indicated groups. Data are presented as mean ± SD. Statistical significance was determined by one-way ANOVA followed by Tukey’s multiple-comparisons test. Exact P values are shown in the figure.

To further assess whether suppression of ferroptosis contributed to the protective effects of sufentanil *in vivo*, we performed a pharmacological reversal experiment using the ferroptosis inducer erastin in the LPS-induced ALI model. Histopathological examination showed that sufentanil pretreatment attenuated LPS-induced disruption of alveolar architecture, inflammatory cell infiltration, and alveolar wall thickening. These protective changes were partially reversed by erastin administration (Figure 6A). Consistently, sufentanil reduced the lung injury score and lung wet-to-dry weight ratio compared with the LPS group, whereas erastin partially reversed these effects (Figures 6B,C). Immunohistochemical analysis showed that 4-HNE immunoreactivity was increased after LPS challenge, reduced by sufentanil pretreatment, and increased again following erastin administration. GPX4 immunoreactivity showed the opposite pattern: it was reduced after LPS exposure, partially restored by sufentanil pretreatment, and subsequently decreased following erastin administration (Figure 6D). Lung MPO activity was also reduced by sufentanil pretreatment and partially restored by erastin administration (Figure 6E). Western blotting and densitometric analysis further showed that sufentanil partially restored GPX4 protein abundance in lung tissues, whereas erastin attenuated this effect (Figures 6F,G). Compared with the LPS + Suf group, the addition of erastin also increased pulmonary Fe^2+^ and MDA levels and reduced the GSH/GSSG ratio (Figures 6H–J). These results show that erastin partially reversed the histological, inflammatory, and ferroptosis-associated protective effects of sufentanil pretreatment in LPS-induced ALI, supporting a contribution of ferroptosis suppression to this protective effect.

## 4 Discussion

Our findings indicate that sufentanil pretreatment attenuates LPS-induced acute lung injury and ferroptosis-associated alterations in both murine lung tissue and A549 cells. GPX4 knockdown markedly weakened the protective effects of sufentanil, whereas erastin partially reversed its protective effects *in vivo*. In addition, the maintenance of GPX4 protein abundance was accompanied by reduced GPX4–LAMP2A colocalization and GPX4–HSC70 association. Collectively, these findings support a model in which sufentanil preconditioning alleviates LPS-induced lung injury, at least in part, by suppressing ferroptosis-associated injury and maintaining GPX4 protein abundance in association with CMA-related lysosomal regulation.

Ferroptosis has increasingly been recognized as a contributor to epithelial, endothelial, and inflammatory injury in ALI and sepsis-associated lung injury (Wen et al., 2024). In the present study, LPS exposure caused iron accumulation, redox imbalance, lipid peroxidation, mitochondrial abnormalities, and reduced GPX4 abundance, whereas sufentanil pretreatment attenuated these changes. These findings support a contributory role for ferroptosis-associated processes in this model rather than merely identifying ferroptosis-related markers secondary to tissue injury. GPX4 is a central antioxidant enzyme that limits ferroptosis by reducing phospholipid hydroperoxides and preserving membrane redox homeostasis. Although GPX4 is commonly discussed in the context of transcriptional regulation, its abundance is also influenced by post-translational mechanisms. For example, ZDHHC20-mediated palmitoylation of GPX4 at Cys66 has been shown to preserve GPX4 protein abundance and increase ferroptosis resistance (Huang et al., 2025). In our study, GPX4 knockdown markedly weakened the ability of sufentanil to improve cell viability and reduce intracellular Fe^2+^, lipid ROS, MDA, and ACSL4 abundance. These loss-of-function findings support a functional contribution of GPX4 to sufentanil-mediated ferroptosis suppression rather than GPX4 serving solely as a parallel marker of cellular protection.

Our results further suggest that CMA-related lysosomal processes are associated with reduced GPX4 protein abundance during LPS-induced injury. GPX4 mRNA expression changed little among the experimental groups, whereas GPX4 protein abundance was markedly reduced by LPS and restored by sufentanil, indicating that the observed protein changes occurred without corresponding detectable transcriptional changes. Chloroquine partially restored GPX4 protein abundance and produced a greater effect than MG-132 under the present conditions, supporting a closer association with lysosome-related processes than with proteasomal turnover. However, pharmacological inhibition alone cannot identify the specific pathway responsible for GPX4 loss because chloroquine can affect multiple lysosome-dependent processes.

Several additional findings support the involvement of CMA-related machinery. CMA selectively targets cytosolic proteins through HSC70-mediated recognition of KFERQ-like motifs and LAMP2A-dependent lysosomal translocation (Kaushik and Cuervo, 2018; Yao and Shen, 2020). Previous studies have linked this machinery to GPX4 degradation and ferroptosis in renal injury, toxic neuronal injury, cancer, and other stress conditions. In our experiments, LPS increased GPX4–LAMP2A colocalization and GPX4–HSC70 association, whereas sufentanil attenuated both changes. LAMP2A knockdown also partially restored GPX4 protein abundance under LPS stimulation. These observations are consistent with previous reports showing that interference with HSC70- and LAMP2A-related processing can limit GPX4 loss. In particular, p23 has been reported to stabilize GPX4 by competing for HSC70 binding and thereby limiting CMA-related GPX4 turnover in non-small cell lung cancer (Chen et al., 2025). Our results extend this line of evidence to an inflammatory ALI context, suggesting that CMA-related lysosomal processes may contribute to the reduction in GPX4 protein abundance during lung injury.

Previous studies have described anti-inflammatory and cytoprotective effects of sufentanil in lung injury and ischemia–reperfusion models (Shi et al., 2024). The present study extends these observations by identifying ferroptosis-associated changes and GPX4 protein abundance as components of sufentanil-mediated protection. Naloxone partially but significantly attenuated the effects of sufentanil on ferroptosis-associated indices *in vitro*. Because naloxone is a nonselective opioid receptor antagonist, these findings support partial opioid receptor involvement but do not establish dependence on a specific receptor subtype (Pugsley, 2002; Heusch, 2015). Residual protection in the presence of naloxone may indicate additional receptor-independent effects, although this possibility remains speculative (Zilka et al., 2017). The partial reversal produced by erastin further supports a contribution of ferroptosis-associated pathways but does not prove a single linear sufentanil–GPX4 pathway.

Several limitations should be acknowledged. First, A549 cells are carcinoma-derived and do not fully reproduce the physiological characteristics of primary alveolar type II epithelial cells. Second, the 40 μM sufentanil concentration used *in vitro* exceeds clinically achievable plasma concentrations and should be regarded as a proof-of-concept dose, with potential off-target effects. Moreover, sufentanil was administered before LPS exposure in both the animal and cell experiments; therefore, the present study demonstrates a preconditioning effect rather than therapeutic efficacy after established ALI. Third, the involvement of CMA-related processes was supported by lysosomal inhibition, GPX4–LAMP2A colocalization, GPX4–HSC70 association, and LAMP2A knockdown. However, other lysosome-dependent pathways, including macroautophagy, cannot be excluded. Direct demonstration of CMA-mediated GPX4 degradation would require protein half-life and degradation-kinetic analyses, mutational validation of candidate KFERQ-like motifs, and direct lysosomal translocation assays (Xu et al., 2020; Kaushi et al., 2015; Wu et al., 2022). Accordingly, our findings should be interpreted as evidence of CMA-associated regulation of GPX4 protein abundance rather than definitive CMA-mediated degradation. Finally, because naloxone is a nonselective opioid receptor antagonist, the present results support only partial opioid receptor involvement and do not establish dependence on a specific receptor subtype.

In conclusion, sufentanil pretreatment attenuated LPS-induced ALI and ferroptosis-associated injury while maintaining GPX4 protein abundance. The accompanying changes in GPX4–LAMP2A colocalization, GPX4–HSC70 association, lysosomal inhibitor responses, and LAMP2A knockdown support the possible involvement of CMA-related lysosomal regulation. However, the present findings do not establish direct CMA-mediated GPX4 degradation or therapeutic efficacy after the onset of ALI.

## Data Availability

The raw data supporting the conclusions of this article will be made available by the authors, without undue reservation.
